# Cancer associated fibroblasts in hematological malignancies

**DOI:** 10.18632/oncotarget.2661

**Published:** 2014-12-06

**Authors:** Lizzia Raffaghello, Angelo Vacca, Vito Pistoia, Domenico Ribatti

**Affiliations:** ^1^ Laboratorio di Oncologia, Istituto G. Gaslini, Genova, Italy; ^2^ Department of Internal Medicine and Clinical Oncology, University of Bari Medical School, Bari, Italy; ^3^ Department of Basic Medical Sciences, Neurosciences and Sensory Organs, University of Bari Medical School, Bari, Italy, National Cancer Institute “Giovanni Paolo II”, Bari, Italy

**Keywords:** cancer associated fibroblasts, mesenchymal stromal cells, hematological malignancies, tumor microenvironment

## Abstract

Tumor microenvironment plays an important role in cancer initiation and progression. In hematological malignancies, the bone marrow represents the paradigmatic anatomical site in which tumor microenvironment expresses its morphofunctional features. Among the cells participating in the composition of this microenvironment, cancer associated fibrobasts (CAFs) have received less attention in hematopoietic tumors compared to solid cancers. In this review article, we discuss the involvement of CAFs in progression of hematological malignancies and the potential targeting of CAFs in a therapeutic perspective.

## Tumor microenvironment

Tumor microenvironment plays an important role in cancer initiation and progression [1]. Individuals affected by chronic inflammatory disorders have an increased risk of developing cancer [2], and, accordingly, treatment with non-steroidal anti-inflammatory drugs reduces tumor incidence [3].

In hematological malignancies, the bone marrow (BM) and the lymph nodes represent the paradigmatic anatomical sites in which tumor microenvironment expresses its morphofunctional features. The lymph node microenvironment is discussed below. BM microenvironment includes hematopoietic stem cells (HSCs) and non-hematopoietic cells. HSCs give rise to all the blood cell types of the erythroid, myeloid, lymphoid and megakaryocytic lineages [4]. The non-hematopoietic cells include endothelial cells, pericytes, fibroblasts, osteoblasts, osteoclasts, macrophages, mast cells, and mesenchymal stem cells [5]. Although a small subset of the latter cells is represented by self-renewing stem cells, the majority of them are multipotential progenitors that can differentiate into osteoblasts, chondrocytes and adipocytes but do not self-renew. Both mesenchymal stem cells and mesenchymal stromal cells are indicated as MSCs.

MSCs contribute to the formation of specialized niches, and namely the endosteal niche closed to the endosteum, and the vascular niche in proximity of the BM vasculature [6]. Quiescent HSCs reside in the endosteal niche, where their interactions is mediated by several factors, including N-cadherins, integrins, Jagged-1, Notch, bone morphogenetic proteins (BMPs), transforming growth factor beta 1 (TGF-β1), angiopoietin-1 (Ang-1), Wnt, and fibroblast growth factor-1 (FGF-1) [7–9]. The vascular niche is a site rich in blood vessels where endothelial cells, pericytes, and smooth muscle cells create a microenvironment that recruits endothelial precursor cells (EPCs), MSCs and HSCs, and is important for stem cell mobilization, proliferation, and differentiation [10–12].

**Figure 1:**
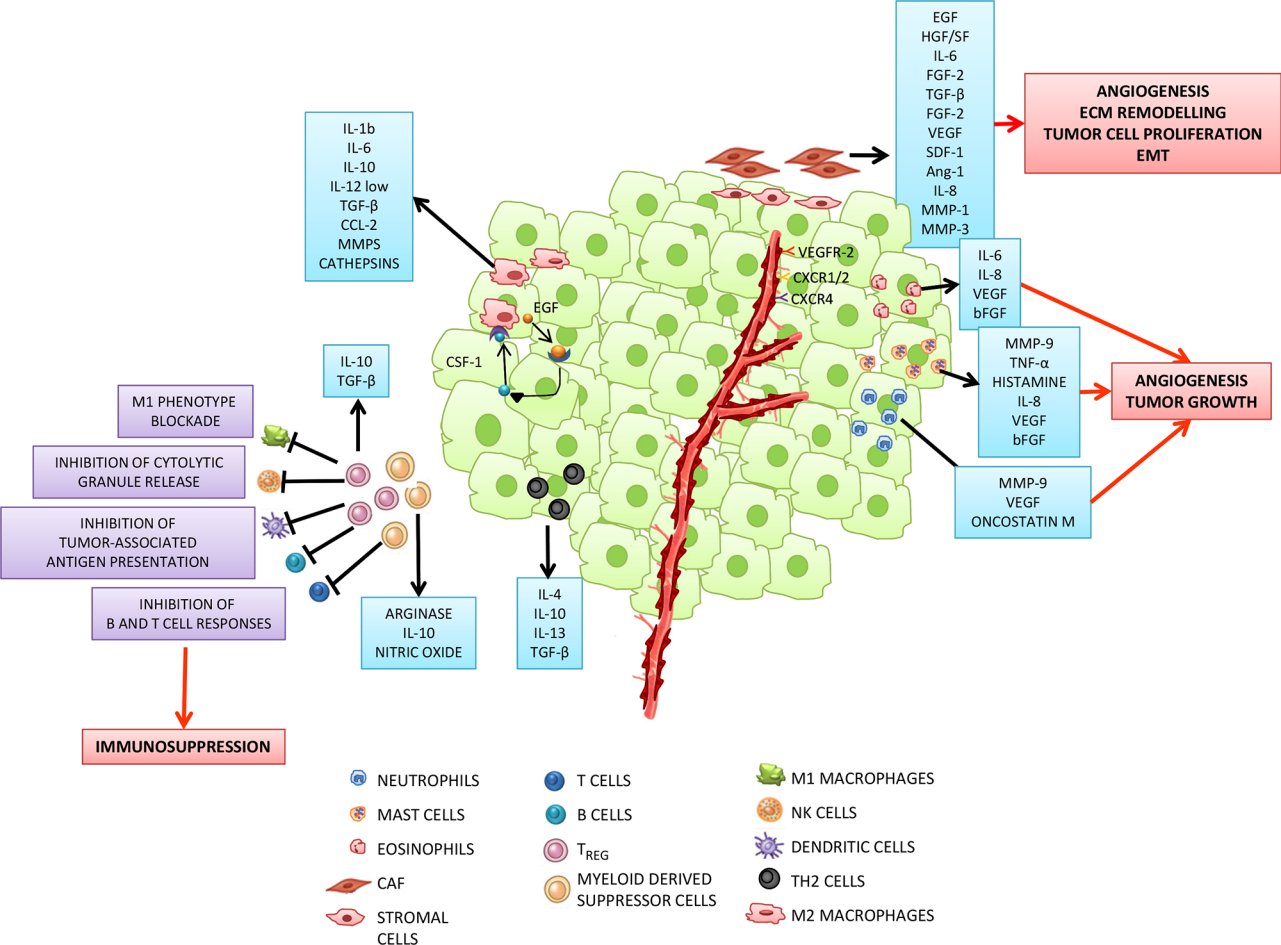
Tumor microenvironment: a complex network of intercellular interactions between tumor and inflammatory cells Cancer cells in primary tumors are surrounded by a complex microenvironment composed of tumor stroma, blood vessels and infiltrating inflammatory cells. Different types of cells are found in the stroma, including fibroblasts, vascular smooth muscle, epithelial and immune cells. The latter cells comprise effectors of both adaptive immunity, such as T and B lymphocytes, and innate immunity, i.e. macrophages, dendritic cells (DCs), neutrophils, mast cells, eosinophils and natural killer (NK) cells. Most of the stromal cells participate in the promotion of the tumor growth. Cancer associated fibroblasts (CAFs) and M2 like polarized macrophages (tumor associated macrophages: TAMs), which can be induced by tumor-derived factors (for example, TGF-β, FGF or PDGF, among others), support tumor growth, angiogenesis, extracellular matrix remodelling and epithelial mesenchymal transition (EMT), by secreting a plethora of pro-tumorigenic proteases, cytokines and growth factors. For example, EGF, secreted by TAMs, participates in a paracrine signaling loop through tumor-secreted colony stimulating factor (CSF-1). VEGF, CXCL12, IL-8 secreted by CAFs or TAMs interact with their respective receptors expressed by endothelial cells and promote tumor angiogenesis. As tumors grow, immune-suppressor cells, including myeloid derived suppressor cells (MDSC) and T regulatory (T_REG_) cells infiltrate the tumor to disrupt immune surveillance through multiple mechanisms, including inhibition of tumor-associated antigen presentation by DCs, T and B cell responses, NK cell cytotoxicity and blockade of M1 macrophage phenotype. Moreover, tumor progression is associated with the increase of TH2 cells secreting immunosuppressive molecules such as IL-4, IL-10 and TGF–β. Mast cells, neutrophils and eosinophils are also recruited to the tumor site where they secrete proliferative and pro-angiogenic factors.

Inflammatory cells in tumor microenvironment communicate via a complex network of intercellular signaling pathways, mediated by surface adhesion molecules, cytokines, and their receptors [13] (Figure 1). Immune cells cooperate with stromal cells as well as malignant cells in stimulating tumor angiogenesis. The latter represents a fundamental mechanism for tumor development and metastatic spread by providing efficient vascular supply.

Among inflammatory cells found in tumor microenvironment, tumor associated macrophages and mast cells support tumor growth and neovascularization by production of a wide variety of angiogenic cytokines, including tumor necrosis factor alpha (TNF-α), TGF-β1, FGF-2, vascular endothelial growth factor (VEGF), platelet derived growth factor (PDGF), interleukin-8 (IL-8), osteopontin and nerve growth factor (NGF). On the contrary, macrophage- and mast cell-derived cytokines that may participate in anti-tumor response include IL-1, IL-2, IL-4, IL-10, and interferon gamma (IFN-γ) [14] (Figure 1).

## Ontogeny of cancer associated fibroblasts

Fibroblasts represent the most prominent cell type of tumor stroma [15]. In the specific context of solid tumors, fibroblasts are referred to as cancer associated fibroblasts (CAFs) and exhibit some similarities with myofibroblasts, originally characterized in wound healing and fibrosis [16, 17]. In breast, prostate, and pancreatic carcinomas, CAFs correlate to high malignancy grade, tumor progression, and poor prognosis [15, 18–20]; in mouse xenografts, CAFs inoculated with carcinoma cells promote tumor cell survival, proliferation and invasive behaviour [21, 22].

**Figure 2:**
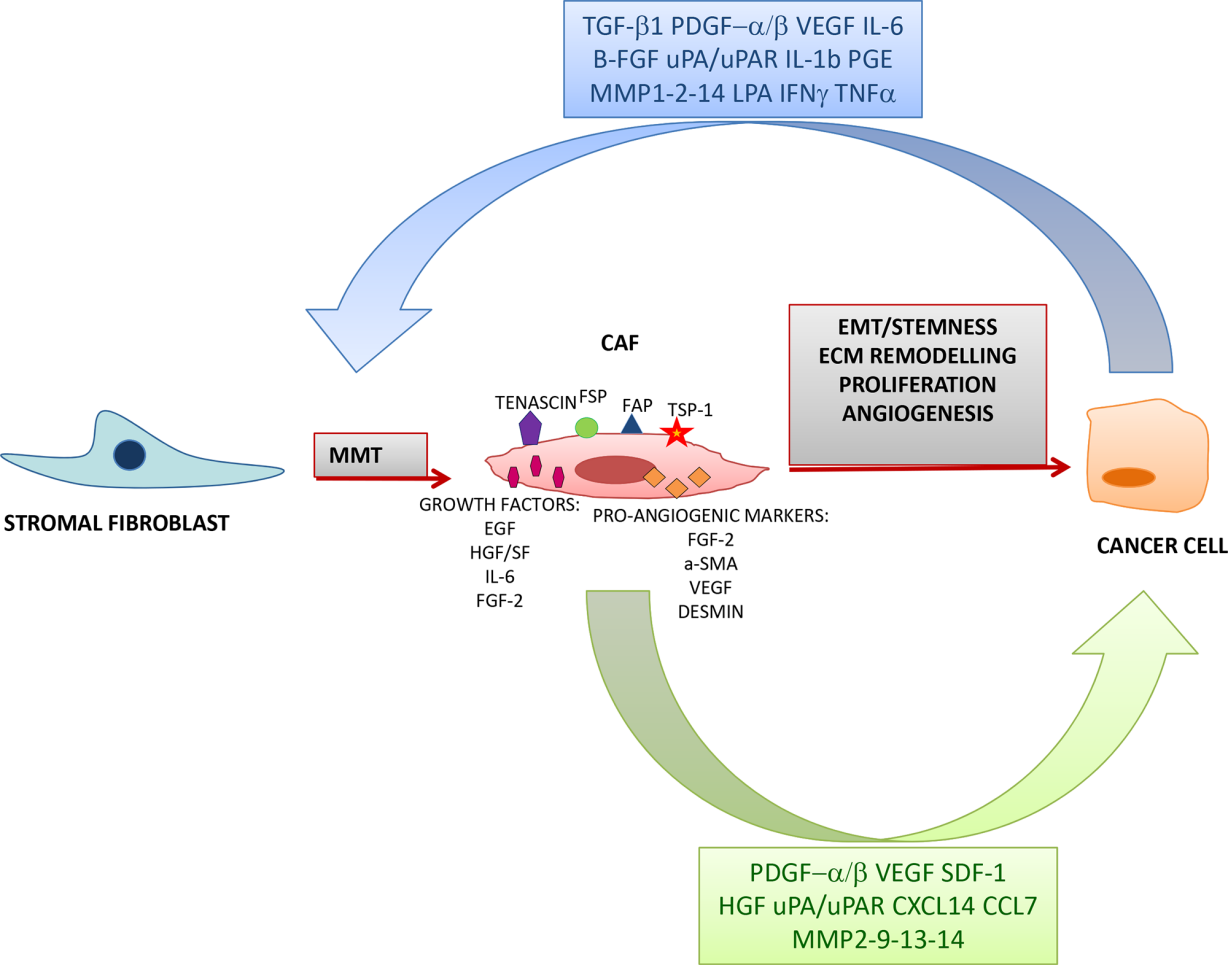
Interaction between CAFs and cancer cells Cancer-derived factors induce a Mesenchymal Mesenchymal Transition (MMT) through which resident normal fibroblast are activated and acquire the expression of various markers such as tenascin, fibroblast specific protein (FSP), fibroblast activation protein (FAP), thrombospondin-1 (TSP-1) and pro-angiogenic molecules. In turn, CAFs secrete various growth factors which sustain tumor progression by promoting Epithelial Mesenchymal Transition (EMT), stemness, extracellular matrix remodelling, proliferation and angiogenesis.

The definition of CAFs encompasses a few important features: i) these fibroblasts are in close contact with cancer cells, ii) CAFs display peculiar immunophenotypic and functional features that sharply differentiate them from normal, resting fibroblasts located in non-neoplastic environments, and iii) the properties acquired by CAFs upon interaction with cancer cells render them supportive of tumor growth and progression through different mechanisms including extracellular matrix remodelling, cell proliferation, angiogenesis and epithelial mesenchymal transition (EMT) [23]. CAF activation is commonly accompanied by the acquisition or upregulation of specific markers which can be classified in four groups: i) the fibroblast activation markers, which include fibroblast specific protein (FSP) and fibroblast activation protein (FAP); ii) the aggressiveness markers thrombospondin-1 (TSP-1), tenascin-C and stromelysin; iii) the pro-angiogenic markers [desmin-1, FGF-2, alpha smooth-muscle-actin (α-SMA) and VEGF]; and iv) the growth factors that support tumor growth and inflammation [Epidermal Growth factor (EGF), Hepatocyte Growth Factor/Scatter Factor (HGF/SF), IL-6, FGF-2] [23, 24]. Moreover, CAFs express matrix-metalloproteinases-1 and -3 (MMP-1, MMP-3), produce collagens, and release cytokines and pro-angiogenic growth factors, including VEGF, HGF, FGF-2, Ang-1, IL-6, IL-8, monocyte chemotactic protein-1 (MCP-1), that have been identified within *in vitro* secretome of adult tissue-derived MSCs [25]. CAFs are a major source of VEGF in VEGF transgenic mice [26], and CAF-derived VEGF enhances the expression and activation of integrins [27]. CAFs release angiogenic factors through proteolysis of the extracellular matrix. In this respect, an elegant study demonstrated that CAFs localized in invasive human breast carcinomas promote tumor growth and angiogenesis through high level secretion of Stromal-Derived-factor-1 (SDF-1)/CXCL12 [22]. Figure 2 summarizes the main features of CAFs and the interactions between CAFs and cancer cells.

**Figure 3:**
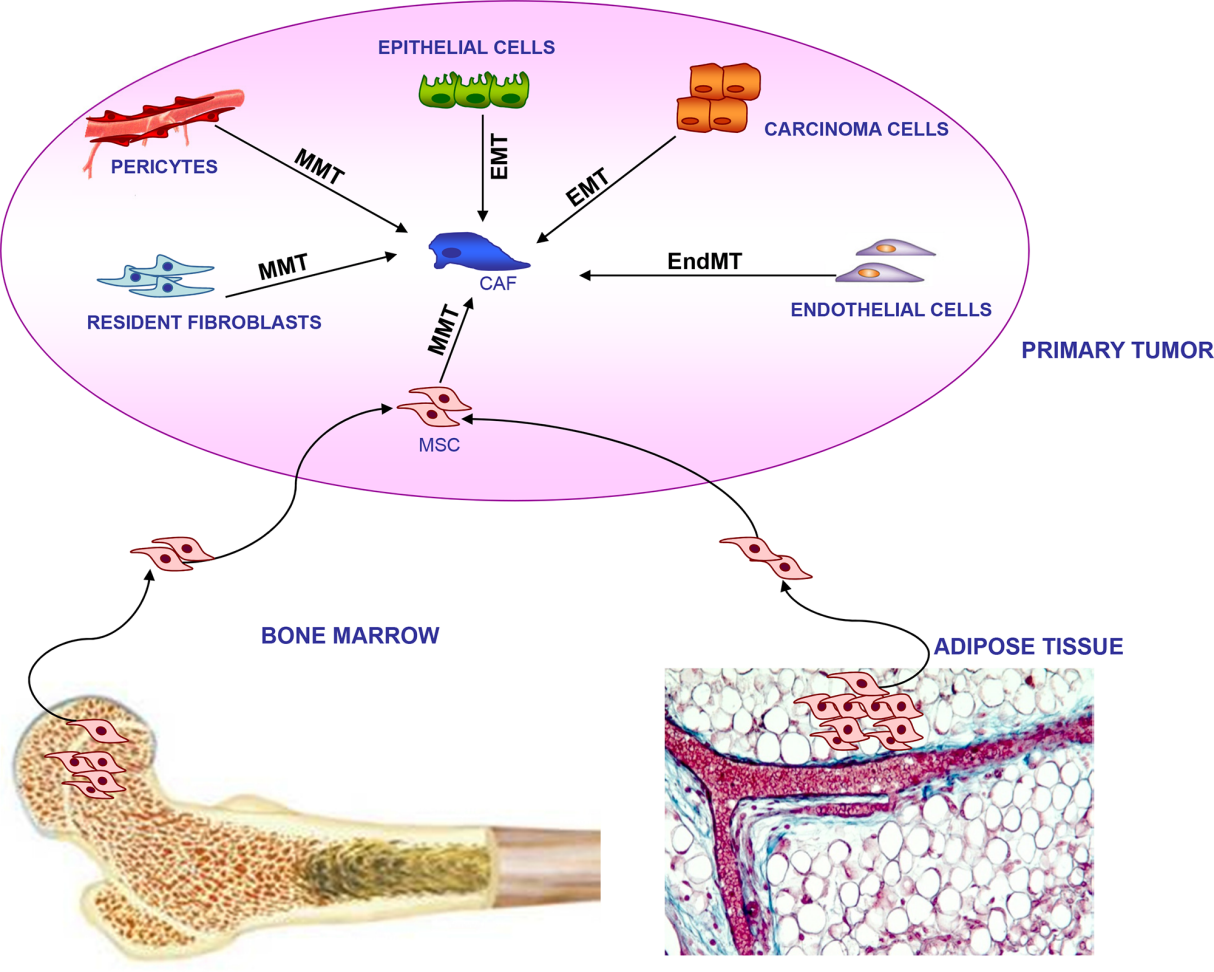
Multiple origin of CAFs within tumor microenvironment CAFs can derive from local cells residing in primary tumor, including epithelial cells and carcinoma cells through Epithelial Mesenchymal Transition (EMT); resident fibroblasts and pericytes through Mesenchymal Mesenchymal Transition (MMT); endothelial cells through Endothelial Mesenchymal Transition (endMT). Alternatively, CAFs may originate from long distance bone marrow and adipose tissue mesenchymal stromal cells (MSCs) through an MMT transition.

The heterogeneity in marker expression led to hypothesize that CAFs could have multiple origins, depending on the tumor histotype and the area of the neoplastic lesion [28]. Indeed, it is now evident that CAFs originate from local or distant reservoirs through different types of transdifferentiation processes as depicted in Figure 3. The primary source is represented by resident fibroblast or pericytes, which differentiate into CAFs through a mesenchymal-mesenchymal transition (MMT) driven by specific cancer-derived factors such as TGF-β, PDGF, FGF-2, and SDF-1 (ref. [29, 30]). In addition, CAFs may originate from local normal or transformed epithelial cells that transdifferentiate through an EMT into activated fibroblasts [31, 32]. These findings were also supported by the observation that stromal cells of breast tumors shared genetic lesions with tumor epithelium [33, 34]. Interestingly, EMT that gives origin to CAFs often occurs in epithelial cells that acquire mutations following oxidative stress [35]. In this regard, an elegant study reported that MMP-3 promotes EMT by inducing the expression of an alternatively spliced form of Rac1, which causes an increase in cellular reactive oxygen species (ROS), and consequently oxidative damage to DNA and genomic instability [35]. On the contrary, other studies demonstrated that somatic mutations can occur exclusively in cancer cells and that CAF mutations are a very rare event [36]. Similarly to EMT, CAFs may also derive from local endothelial cells through an endothelial mesenchymal transition (EndMT) mainly driven by tumor-derived TGF-β. During this process, endothelial cells loose the expression of CD31 and acquire that of mesenchymal markers like α-SMA and FSP (ref. [37]).

BM and adipose tissue (AT) represent two distant sites that significantly contribute to CAF generation [28]. In particular, cancer-derived soluble factors recruit BM- and AT-derived MSCs to tumor sites, where the latter cells acquire the expression of CAF-specific markers such as α-SMA, FAP, tenascin-C and TSP-1 [38, 39]. More importantly, in tumor microenvironment MSCs exhibit typical functional properties of CAFs, including high expression of SDF-1 and the ability to promote tumor cell growth both in *in vitro* models and in *in vivo* coimplantation assays [40]. It is of note that most of the studies regarding CAF origin were performed in mouse tumors. In contrast, information from human tumors is scarce.

## Differences and similarities between CAFs and MSCs

The definition of CAFs, that has become popular for most solid tumors, is rarely utilized to indicate stromal cells of mesenchymal origin found in the specific microenvironments of hematopoietic malignancies, i.e. BM and lymph nodes. In these disorders, such stromal cells are often referred to as mesenchymal stromal cells, that, in analogy to mesenchymal stem cells, are indicated as MSCs. However, as it will be discussed below, the few comparisons that have been done between leukemia-associated MSCs and classical solid tumor-related CAFs have demonstrated that these cell types share many phenotypic and functional features. Thus, for practical reasons, the terms CAF and MSC will be used interchangeably, the latter indicating MSCs generated following *in vitro* culture.

The primary functions of fibroblasts are the synthesis and deposition of collagen that builds up the scaffold of connective tissues [16, 23]. CAFs acquire a new functional polarization translating into production and release of proteases that digest the extracellular matrix (e.g. MMPs), pro-angiogenic factors that stimulate microvessel formation and pro-metastatic molecules that accelerate tumor cell dissemination to distant sites [23].

MSCs have a similar spectrum of activities but, in contrast to fibroblasts, they have been extensively investigated for their immunoregulatory properties [41, 42]. In detail, MSCs selectively alter immune cell functions by suppressing T cell activation or denditric cells or by inhibiting the cytotoxic capacity of NK cells. Nonetheless, the few studies that have addressed the immumodulatory activities of classic fibroblasts of mesodermal origin have shown that the latter behave as MSCs [43, 44]. Moreover, MSCs may differentiate along osteogenic, adipogenic, and chondrogenic lineages [45]. Finally, MSCs express stromal cell markers (CD73, CD105, CD44, CD 29, and CD90) in the absence of hematopoietic and endothelial markers [46].

## Fibroblasts and hematological malignancies

As mentioned in the introductory section of this article, most of the published studies addressing the role of stromal cells in leukemia and lymphoma microenvironments have been carried out using MSCs expanded *in vitro* for a few passages before being tested. On the other hand, since MSCs are the precursors of fibroblasts, it is conceivable that *bone fide* fibroblasts have similar functional properties.

This conclusion is supported by the few studies focused on BM fibroblasts from patients with hematological malignancies. Frassanito and co-workers isolated primary BM fibroblasts from multiple myeloma (MM) patients (see below) and showed that these cells have multiple features of CAFs and act to promote tumor growth [47]. Hairy cell leukemia (HCL) is a chronic B cell malignancy characterized by a progressive fibrotic process mainly due to the accumulation of fine argyrophilic reticulin fibers that, in advanced stage patients, may coexist with collagen fibers [48]. The glycoprotein fibronectin, that is produced and assembled by HCL cells, contributes to the fibrotic process in the BM of HCL patients, which is enhanced by HCL cell-derived FGF-2 [49]. These findings demonstrate that HCL-associated BM fibrosis results from accumulation and assembly of collagenous (reticulin) and non-collagenous (fibronectin) extracellular matrix components. Fibroblastoid cells closely associated with collagen fibers synthesize retic ulin and collagen in HCL BM, but no significant increase in fibroblast numbers is observed at the latter site [50]. Collectively, these observations suggest that BM fibroblasts are activated by the BM microenvironment to synthesize reticulin and collagen rather than to proliferate and expand, as instead occurs in the BM of MM patients. Indeed it was shown that TGF-β1 produced predominantly by HCL cells enhanced the production and deposition of reticulin and collagen fibers by BM fibroblasts. TGF-β1 was detected at high concentrations in BM, serum and plasma from HCL patients, and active TGF-β1 correlated with the extent of BM fibrosis and infiltration with HCL cells. Other studies showed that adhesion of HCL cells to hyaluronan stimulated their production of FGF-2 and fibronectin, the latter through the v3 isoform of CD44. Since HCL cells express also the receptor for FGF-2, autocrine production of the latter cytokine is the main responsible for fibronectin production. Thus, FGF-2 plays a relevant role in the pathogenesis of BM fibrosis, as also supported by the immunohistochemical detection of large amounts of FGF-2 in fibrotic BM [51].

In conclusion, the above studies, performed a decade or more ago, have demonstrated that BM fibroblasts are activated by HCL cell-derived TGF-β1 and FGF-2 to produce and deposit reticulin and collagen fibers, thus causing the peculiar BM fibrosis of HCL. HCL cell-activated fibroblasts represent a typical example of CAF, but this definition was not yet in use when these results were published.

Before reviewing the relationships between MSCs and tumor cells from specific hematological malignancies, a few general considerations can be anticipated. First of all, MSCs establish mutual interactions with cancer cells whereby both cell types undergo profound changes in their activation state, proliferative potential, pro-angiogenic and migratory activities, and extracellular matrix remodeling ability. MSCs consistently support survival of tumor cells and shield them from the cytotoxic effects of chemotherapy by creating a protective niche. MSCs may also stimulate the proliferation and expansion of malignant cells. On the other hand, tumor cells modify MSCs, for example by enhancing their proliferation and chemokine production. Finally, tumor cell-conditioned MSCs modulate the functions of other non-malignant cells present in the tumor microenvironment shifting them towards a tolerogenic and immunosuppressive phenotype. The latter cells, in turn, may influence the phenotype and function of MSCs. All of the above cell-to cell interactions occur through both contact-dependent and soluble factor-dependent mechanisms. These interactions involve signal transduction and metabolic pathways that vary in the individual hematological malignancies and have been characterized only partially. Elucidation of such pathways may represent a promising avenue to the development of novel therapeutic strategies targeting the mesenchymal stroma in the tumor microenvironment.

A final general issue that deserves discussion is the developmental relationship between MSCs and malignant cells of hematopoietic lineage. Studies performed predominantly with MSCs isolated from the BM of patients with acute lymphoid or myeloid leukemias or MM have demonstrated that MSCs are often genomically unstable and carry cytogenetic alterations that may be related or unrelated to those detected in autologous tumor cells [52–54]. In the former case, the identity of genetic abnormalities in cancer cells and paired MSCs may be explained by the occurrence of cell fusion with transfer of genetic material, the origin of tumor cells and MSCs from a common progenitor/stem cell, or the differentiation of a subset of tumor cells into MSCs [53, 54]. However, so far, none of the above hypotheses has been experimentally proven. Occurrence in MSCs of cytogenetic abnormalities unrelated to those found in autologous tumor cells may be related to molecules released in the tumor microenvironment capable of damaging DNA, such as oxygen or nitrogen radicals. In this respect, cytogenetically abnormal endothelial cells have been detected in pre-clinical solid tumor models [55]. Box 1 contains a schematic definition of haematopoietic malignancies reviewed below.

Box 1 **Principal hematological malignancies**

**ACUTE LEUKEMIAS**

Acute leukemias are hematological malignancies originating from early hematopoietic progenitors/precursors and involving the myeloid (acute myeloid leukemia or AML) or the lymphoid (acute lymphoid leukemia or ALL) lineages. Acute leukemias, that can be further subclassified according to cytogenetic, morphological or immunophenotypic criteria, develop initially in the bone marrow, then invade the circulating compartment and metastasize to distant organs.

**CHRONIC LYMPHOCYTIC LEUKEMIA (CLL)**

CLL is a monoclonal expansion of CD19+, CD5+ B lymphocytes carrying either mutated or unmutated immunoglobulin variable region (IgV) genes. Mutated cases have a better prognosis than unmutated cases, which are also characterized by the expression of the CD38 and ZAP-70 markers. CLL, that often manifests as asymptomatic blood lymphocytosis, develops in the lymph node and bone marrow microenvironments.

**FOLLICULAR LYMPHOMA (FL)**

FL is a B cell malignancy that develops in secondary lymphoid follicles and contains centroblasts and centrocytes in variable proportions. Tumor cells may invade the bone marrow and circulate in the peripheral blood. FL is an indolent lymphoma characterized by the progressive infiltration of lymphoid follicles with tumor cells expressing constitutively the anti-apoptotic protein Bcl-2. Invaded lymph nodes may retain for a long time remnants of their physiological architecture.

**HODGKIN LYMPHOMA (HL)**

HL is a peculiar germinal-center derived B cell malignancy that does not express B cell markers and is characterized by a paucity of tumor cells known as Reed-Sternberg cells. HL, that develops in the lymph node microenvironment, is classified in two clinicopathologic entities, namely classical HL (~95% of cases) and nodular lymphocyte predominant HL (~5% of cases). HL lymph nodes are enriched with various reactive cell types, such as eosinophils, neutrophils, plasma cells and T cells.

**MULTIPLE MYELOMA (MM)**

MM is monoclonal plasma cell malignancy that develops and grows in the bone marrow, and metastasizes to the bone.

## BM-MSCs in acute leukemia

BM-MSCs have been isolated and expanded *in vitro* from patients with acute lymphoblastic leukemia of B cell lineage (B-ALL) or acute myeloblastic leukemia (AML) before being characterized in different studies as to functional and genetic features.

B-ALL cells, as their normal counterparts represented by early-B cells, depend on close interactions with the BM microenvironment for *in vitro* survival and growth [56–61]. Some of the early studies performed on this issue made use of mouse fibroblasts or fibroblast cell lines as sources of stromal cells interacting with leukemia cells.

BM-MSCs were shown to inhibit the *in vitro* proliferation of acute leukemia cells by inducing the transient arrest of malignant cells in the G1 phase of the cell cycle. In contrast, when acute leukemia cells were injected in immunodeficient mice in combination with BM-MSCs, the latter cells were found to accelerate tumor growth, possibly through the formation of a cancer stem cell *niche* in which leukemic cell proliferation is supported and stimulated [62].

BM-MSCs express the Notch ligands Jagged-1/2 and Delta ligands (DLL-1/4) that interact with Notch receptors 1–4. Upon B-ALL cell co-culture with BM-MSCs, inhibition of Notch signaling resulted into reduced survival of leukemic cells. In particular, inhibition of Notch-3 and -4 or Jagged -1/2 and DLL1 caused massive apoptosis of cultured B-ALL cells, indicating that these molecules are synergistically involved in the stromal cell-mediated antiapoptotic effect [63].

The *niche* concept applies also to another property of BM-MSCs that has been extensively characterized, i.e. their ability to protect cancer cells from the effects of anti-neoplastic drugs through different mechanisms and therefore to exert a pro-survival effect.

One of the therapeutic strategies for B-ALL is the administration of L-asparaginase that depletes leukemic cell of asparagine, thus promoting death of malignant cells. It was shown that co-culture of B-ALL cells with MSCs protects the former cells from the cytotoxic effect of L-asparaginase since MSCs synthesize high amounts of L-asparagine (approximately 20 times higher than those detected in B-ALL cells) and transfer this aminoacid to tumor cells, rendering them resistant to the drug [64]. More recently, a mechanism involved in protection of ALL cells from L-asparaginase and operated by adipocytes, i.e. mature mesenchymal cells deriving from MSCs, has been identified. It was shown that, in children treated for high risk ALL, glutamine synthetase was strongly increased in BM adipocytes after induction chemotherapy. Adipocytes are a major source of glutamine; therefore they were co-incubated *in vitro* with leukemic cells and found to protect the latter cells from the toxic effects of L-asparaginase through a glutamine-dependent mechanism [65].

MSC-driven protection of leukemic cells extends to numerous chemotherapeutic agents. Examples of the molecular interactions involved are represented by the CXCR4/CXCL12 axis and the VLA-4/VCAM1/CD44 adhesion molecules.

Stromal cells produce CXCL12 that attracts CXCR4^+^ leukemic cells and protects them from cytotoxic agents by reducing the activation of caspase-3 and enhancing the expression of anti-apoptotic proteins as Bcl-XL. This has been elegantly demonstrated in chronic myeloid leukemia (CML) cells exposed to imatinib in the presence or absence of MSCs, leading to the proposition of combination therapy with imatinib and the CXCR4 antagonist AMD3100 to overcome MSC-mediated chemoresistance [66]. Another study focused on CML cells demonstrated that imatinib induces up-regulation of CXCR4 on leukemic cells, thus increasing their migration to the BM stroma that produces CXCL12. The final result of these cellular interactions is the increased survival of quiescent CML cells [67].

Integrins trigger intracellular signaling pathways by forming macromolecular complexes with numerous plasma membrane proteins including ion channels. ALL blasts, upon co-culture with BM-MSCs, upregulated the expression of a molecular complex comprised of hERG-1 channels, the β1 integrin subunit and CXCR4, that activates the ERK1/2 and PI3K/Akt signaling pathways and promotes chemoresistance [68]. The Wnt pathway has been shown to be involved in MSC-mediated drug resistance of ALL cell lines. ALL cells, upon co-culture with MSCs, up-regulated the expression of various components of the Wnt pathway, and inhibition of the latter sensitized leukemic cells to chemotherapy [69].

The MLL-AF4 gene fusion is a peculiar genetic abnormality detected in infant acute pro B-ALL, that is known to arise *in utero* [70]. It was shown that, out of thirty eight B-ALL patients with different fusion genes studied, only those cases carrying the MLL-AF4 fusion in leukemic blasts displayed the same genetic translocation detected in autologous BM-MSCs. However, at variance with leukemic blasts, BM-MSC did not display monoclonal rearrangements of immunoglobulin (Ig) genes. These findings rule out that the possibility that leukemic blasts underwent cellular plasticity or dedifferentiation to MLL-AF4^+^ MSCs, but are consistent with the hypothesis that the tumor cells and MSCs originated from a common mesodermal precursor. Notably, MLL-AF4^+^ MSCs were euploid, thus precluding the possibility of cell fusion, and did not show any proliferative advantage in culture [53].

Different results were obtained in a smaller study of ten B cell precursor ALL cases carrying three different chromosomal translocations (TEL-AML1, E2A-PBX1 and MLL rearrangement, respectively). In all cases, variable proportions of BM-MSCs were found to display the same chromosomal translocation as autologous leukemic blasts. Furthermore, BM-MSCs from three patients displayed the same monoclonal Ig rearrangement detected in autologous tumor cells, indicating a clonal relationship between stromal and leukemic cells. Two possible models can account for such relationship, i) the existence of a common progenitor cell carrying leukemia-specific chromosomal translocation and monoclonal Ig gene rearrangement and capable of differentiating into leukemic or stromal cells, and ii) the de-differentiation of leukemic cells into MSCs displaying the same genetic abnormality and Ig rearrangement [54].

More recently, BM-MSCs were isolated from 45 pediatric patients with ALL and subjected to genetic and functional investigations. Reduced proliferative capacity and ability to support *in vitro* hematopoiesis were observed in BM-MSCs from leukemic patients compared to BM-MSCs from control subjects. However, no cytogenetic abnormality was ever detected in the former or the latter cells [71]. Accordingly, BM-MSCs from ALL and chronic lymphocytic leukemia (CLL, see below) patients had a normal karyotype, and aneuploid BM-MSCs were detected only in cases carrying chromosomal abnormalities [72].

Cytogenetic studies have been performed also with BM-MSCs from patients with myeloproliferative neoplasms (MPN) including myelodysplastic syndrome (MDS), AML and Philadelphia negative MPN. Blau and coworkers reported that chromosomal aberrations were present in 15/94 MDS/AML patients tested; such abnormalities were consistently different from those detected in paired malignant hematopoietic cells, indicating the absence of any clonal relationship between stromal and tumor cells. These Authors identified a correlation between BM-MSC genetic aberrations and overall survival in their cohort of patients, but raised a note of caution since these aberrations were detected only in cases with adverse prognosis and therefore were not likely to represent an independent prognostic factor [52]. Nonetheless, the possibility that genetic instability of BM-MSCs may translate into the support to MDS/AML growth is consistent with pre-clinical studies [73]. In this connection, an intriguing study of two AML cases with MLL-ELL translocations showed that non-adherent leukemic blasts, upon long-term culture, transformed into myofibroblasts capable of supporting the *in vitro* growth of autologous leukemic cells [74].

Avanzini et al. have recently reported that BM-MSC from five MPN patients out the 23 tested displayed genetic abnormalities unrelated to those detected in transformed myeloid cells. In addition, patient BM-MSCs displayed several functional defects when compared to BM-MSCs from healthy donors that led the Authors to postulate a role of abnormal stromal cells in leukemogenesis [75].

In conclusion, i) BM-MSCs from patients with acute lymphoid or myeloid leukemia promote survival and growth of tumor cells as well as chemoresistance, and may be functionally abnormal and ii) genetic studies have delineated complex developmental relationships between BM-MSCs and acute leukemia cells, with heterogeneous results from different studies that do not yet allow to delineate a comprehensive model.

## BM-MSCs in chronic lymphocytic leukemia (CLL)

Studies performed with BM-SCS from CLL patients have generated results fully consistent with those obtained in acute leukemia patients, i.e. stromal cells promote survival, migration and chemoresistance of leukemic cells [76, 77]. However, CLL studies have led to the identification of novel and perhaps tumor cell-“specific” mechanisms of interplay between malignant cells and stromal cells.

Ding *et al* have demonstrated that CLL cell supernatants induce Akt activation and proliferation in MSCs through PDGF receptors triggering. Accordingly, two PDGFR ligands, i.e. PDGF and VEGF, were detected in CLL supernatants, but PDGF only was found to be crucial for MSC activation *via* a PI3K-dependent mechanism [78].

Primary CLL cells were shown to possess a limited ability to transport cystine for glutathione synthesis due to the low expression of the Xc transporter. BM-MSCs, in contrast, are very efficient at importing cystine and converting it to cystein, that is released in the tumor microenvironment and uptaken by CLL cells to promote glutathione synthesis. Availability of high concentrations of the latter molecule promotes survival of CLL cells and protects them from the toxicity of chemotherapeutic agents. Inhibition of the stroma-driven protective mechanism sensitized leukemic cells to cytotoxic drugs even in the presence of stromal cells. Targeting this biochemical pathway may represent a promising therapeutic avenue [79].

Another study has led to the identification of a novel survival signaling pathway activated in stromal cells by contact with primary CLL cells. It was shown that induced expression of PKC-β in BM-MSCs was indispensable for the capability of the latter cells to support CLL survival. Mice knocked out for PKC-β expression were refractory to CLL transplantation, highlighting the relevance of this novel mechanism *in vivo* in the tumor microenvironment. Two points are worth mentioning, i) part of the *in vitro* experiments were carried out using the murine stromal cell line EL08-1D2, that displayed the typical characteristics of CAFs. Such experiments were then confirmed with BM-MSCs isolated from CLL patients, that also displayed most CAF features. ii) Expression of PKC-β was found to be high in biopsies from CLL, ALL and mantle cell lymphoma patients, pointing to a mechanism activated in different lymphoid malignancies [80].

Finally, a biochemical mechanism for resistance of CLL cells to forodesine has been identified. Leukemic cell apoptosis induced by forodesine was significantly inhibited in the presence of human or mouse MSCs. Forodesine-induced dGTP depletion and GTP and ATP accumulation in tumor cells were inhibited by MSCs together with RNA and protein synthesis. Furthermore, MSCs upregulated in leukemic cells the expression of the anti-apoptotic protein Mcl-1 [81].

## BM-MSCs and lymphoma

The role of MSCs/fibroblasts in the micro-environment of classical Hodgkin lymphoma (cHL) and follicular lymphoma (FL), two malignancies originating from germinal center B cells, will be reviewed.

cHL is composed of mononuclear Hodgkin cells and multinucleated Hodgkin and Reed-Sternberg (HRS) cells embedded in an abundant microenvironment that is populated by different non-neoplastic cell types, including B and T cells, plasma cells, eosinophils, mast cells and histiocytes/macrophages. Fibroblast-like cells and interdigitating reticulum cells are present in large numbers in the collagen bands of nodular sclerosis cHL, where they are frequently associated with HRS cells [81].

Although recruitment of non-malignant cells to the cHL microenvironment is largely mediated by tumor cells, the former cells activated by the latter can contribute to the phenomenon. Thus, for example, HRS cells, that do not produce eotaxin, can induce *in vitro* production of this chemokine by dermal fibroblasts through the release of IL-13 and TNF [82].

cHL-derived fibroblasts express stem cell factor (SCF) as both membrane and soluble molecules that bind to c-kit expressed on HRS cells thereby activating the latter cells in a paracrine way [83]. HRS growth and survival are supported also by other molecules produced by stromal cells such as IL-7 and CCL5. On the other hand, HRS cells themselves produce IL-7 that induces the synthesis of IL-6 in HL-derived fibroblasts [84]. Altogether, these studies demonstrate that fibroblasts/stromal cells play a relevant role in cytokine-mediated cross-talk among HRS and other cells types, culminating in stimulation of tumor cell survival and growth.

The FL microenvironment contains different types of immune cells (Treg cells, T follicular helper cells, macrophages) admixed with cells MSCs. In normal lymph node, three different subsets of MSCs have been identified and characterized, i) fibroblastic reticular cells (FRC) that, by producing CCL9, CCL21 and CXCL12, attract mature dendritic cells, naïve T and B cells and promote their interactions in the T cell zone, ii) follicular dendritic cells (FDCs), that attract B cells in the light zone of the germinal center by producing CXCL13 and participate in the selection of high affinity B cells, and iii) marginal reticular cells, that deliver small antigens to B cells through follicular conduits [85]. The FRC network appears to be activated in FL lymph nodes [86] and FDCs display an undifferentiated phenotype [87]. Both FRCs and FDCs contribute to establish a protective niche for FL cells where the latter cells survive and grow. A similar, ectopic niche develops in the BM, that is often infiltrated by FL cells.

Stromal cells support FL cell recruitment and growth by a combination of adhesion molecules (e.g. VCAM-1) and chemokines/cytokines (e.g. CXCL-12, CXCL-13, BAFF, HGF, IL-15). These processes are favored by TNF and LTα1β2, that are overexpressed by FL cells and promote lymphoid stromal cell differentiation. FL-associated MSCs overexpress also the chemokine CCL2, that attracts monocytes and stimulates their polarization towards a tumor-associated macrophage (TAM) phenotype. Finally, TFH cells overexpress TNF and LTα1β2 that triggers differentiation of stromal cells into FRCs. Similar mechanisms operate in LN and BM from FL patients (as reviewed by Thomazy et al. [86]) and FDCs display an undifferentiated phenotype [87].

## CAFs and multiple myeloma

Frassanito et al. [47] identified CAFs expressing FSP1, αSMA, and FAP in BM samples of patients with MM or monoclonal gammopathies of undetermined significance (MGUS). The highest proportions of CAFs were found in active MM patients. Immunohistochemistry of patients' BM confirmed that high numbers of CAFs coexist with MM cells. The CAF population was heterogeneous since it expressed cell markers restricted to endothelial cells, HSPCs, and MSCs, which implies their multiple cell derivation. Moreover, Frassanito et al. [47] demonstrated that MM activate fibroblasts and recruit them via secretion of TGF-β. CAFs, in turn, transform BM stroma by producing collagen and fibronectin, and by secreting growth factors (TGF-β, HGF, IGF1), cytokines (IL-1, IL-6) and chemokines (SDF-1α) [15]. TGF-β and conditioned media from MM cells and active MM CAFs converted patients' MM endothelial cells and HSPCs into CAF-like cells. Frassanito et al. [47] using the *in vivo* xenograft MM 5T33 mouse model, demonstrated that animals co-injected with active MM CAFs and MM cells showed faster tumor growth than those injected with MM cells alone. Finally, Frassanito et al. [47] found that inhibition of the SDF1α/CXCR4 axis affects the MM cell migration, adhesion and proliferation indicating that MM CAFs recruit CXCR4^+^ MM cells *via* SDF1-α secretion.

**Figure 4:**
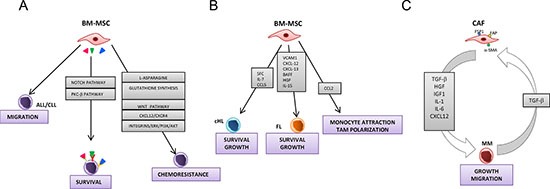
Models for the role of bone marrow-mesenchymal stromal cells and cancer activated fibroblasts in hematological malignancies Panel A depicts the interactions between bone marrow-mesenchymal stromal cells and acute lymphocytic leukemia (ALL) and chronic lymphocytic leukemia (CLL) cells. BM-MSCs promote migration, survival and chemoresistance of ALL/CLL cells. Induction of survival by BM-MSCs is mediated by different pathway including Notch and PKC-β. BM-MSC protect ALL/CLL cells from cytotoxic agents through various mechanisms such as the synthesis of L-asparagine and glutathione, and the involvement of Wnt, ERK 1/2, PI3K/Akt signaling pathways and CXCR4/CXCL12 axis. Panel B shows the interactions between BM-MSCs and classical Hodgkin lymphoma (cHL) and follicular lymphoma (FL) cells. BM-MSCs support the growth and survival of lymphoma cells by different molecules including stem cell factor (SCF), IL-7, IL-15, CCL5, CXCL-12, CXCL-13, BAFF, Hepatocyte growth factor (HGF). FL-associated MSCs overexpress CCL2 that attracts monocyte and stimulates their polarization towards a tumor-associated macrophage (TAM) phenotype. Panel C depicts the interplay between multiple myeloma (MM) cells and CAFs. MM activate and recruit fibroblasts via the secretion of TGF-β. In turn, CAFs secrete different growth factors and cytokines that stimulate growth and migration of MM cells.

In conclusion, BM-MSCs/CAFs promote growth, survival and migration of cells derived from haematological malignancies by different pathways as depicted in Figure 4.

## Therapeutic implications

Given their relevant role in driving tumor progression, CAFs have recently emerged as therapeutic targets by various strategies. The interplay between CAFs and cancer cells is characterized by a feed-forward loop in which various growth factors/cytokines and their receptors play an important role in promoting tumor growth and progression. If on one side tumor cells secrete a plethora of factors such as TGF-β1, PDGF, VEGF, IL-6, FGF-2, IFN-γ, TNF, MMPs involved in fibroblast activation, on the other side CAFs produce the same molecules that in turn affect tumor aggressiveness. In this context, several strategies have been designed to target the growth factors and their receptors by the use of antagonists or specific antibodies that, in some cases, may act simultaneously on tumor cells and CAFs. For example, the recent FDA approved pazopanib (Votrient), a multi target receptor tyrosine kinase inhibitor (TKI) against PDGFR α/β, VEGFR-1, -2, -3 and c-kit [88], as well as MET/HGF inhibitors (monoclonal antibodies against HGF and selective MET tyrosine kinase inhibitors) [89] and the humanized monoclonal antibody (mAb) against VEGF-A Bevacizumab (Avastin) [90] have been validated to target the same molecules expressed by both tumor cells and CAFs. Particular interest has been focused on specific blockers of PDGF and HGF receptor signaling in CAFs, that proved to inhibit tumor growth and progression in preclinical tumor models [91–93].

Another approach has been designed to target the urokinase plasminogen activation (uPA) uPA/uPA receptor (R) system that represents one of the key systems driving tumor invasion and metastases [94]. uPAR, overexpression in cancer cells and tumor-associated stromal cells such as CAFs, is associated to poor prognosis and in some cases is predictive of invasion and metastasis [95, 96]. Thus, new uPAR-targeted therapies might be effective against both tumor cells and cells of the tumor microenvironment. Several therapeutic approaches aimed at inhibiting the uPA/uPAR, such as selective inhibitors of uPA activity, antagonist peptides, mAb able to prevent uPA binding to uPAR and gene therapy techniques silencing uPA/uPAR expression functions, have been shown to possess anti-tumor effects in xenograft models [97]. However, all these strategies need definitive confirmation in humans as only few uPA inhibitors entered clinical trial.

Another interesting approach concerns the use of inhibitors of COX-2, an inflammatory molecule overexpressed in both CAFs and tumor cells upon their reciprocal interaction [98, 99]. COX-2 promotes tumor progression by inducing EMT and stimulating VEGF-mediated angiogenesis and MMP-14-driven invasion [84, 100]. In this respect, preclinical and clinical trials demonstrated that COX-2 inhibitors such as Celecoxib (Celebrex) represent a promising strategy in the prevention and treatment of solid tumors [101].

Given the relevant role of MMPs in promoting tumor invasiveness and metastasis, great efforts have been undertaken in order to develop specific MMP inhibitors [102]. The most extensively studied classes of MMP inhibitors include Batimastat, Marimastat, Prinomastat and Tanomastat that have been tested in clinical trials for several malignant diseases [103].

FAP, a member of the serine protease family, selectively expressed on CAFs and cancer cells, exerts a proteolytic activity that supports tumor growth and proliferation [104]. Thus, FAP has been considered as an emerging therapeutic target in cancer. A humanized anti-FAP antibody (mAb F19; sibrotuzumab) was well tolerated [105] but failed to show any beneficial effect in a phase II trial for metastatic colorectal cancer [106]. It was reported that a monoclonal anti-FAP antibody conjugated to maytansinoid induced a long-lasting inhibition of tumor growth and complete regression in different experimental tumor models, without signs of toxicity [107]. However, further studies are required to determine the potential clinical application of this molecule.

An interesting approach to target CAFs focuses on the development of specific antibodies against integrins, that have been found to be expressed by tumor cells and CAFs and to be involved in cancer progression [108]. In particular, a recent study demonstrated that integrin αvβ6 triggering leads to CAFs proliferation, and consequently increases gastric cancer metastasis [109]. Furthermore, a human therapeutic antibody 264RAD, which binds to αvβ6 and inhibits its function, has been shown to delay tumor growth by preventing TGF-β-mediated activation of CAFs, and by reducing the expression of fibronectin and α-SMA on stromal fibroblasts [110]. Therefore, disruption of the expression of integrin αvβ6 may be a new strategy for future cancer therapy.

Finally, curcumine can interfere with the dynamic mutual interaction between CAFs and head and neck tumor cells [111]. Specifically, curcumin reduced the release of EMT-mediators by CAFs with consequent reduction of cancer invasion. These data confirmed the potential of curcumin in clinical application and underline the need of improved formulation for *in vivo* delivery.

## References

[1] Balkwill F, Mantovani A (2001). Inflammation and cancer: back to Virchow?. Lancet.

[2] Balkwill F, Charles KA, Mantovani A (2005). Smoldering and polarized inflammation in the initiation and promotion of malignant disease. Cancer Cell.

[3] Koehne C-H, Dubois RN (2004). COX-2 inhibition and colorectal cancer. Semin Oncol.

[4] Krause DS (2002). Regulation of hematopoietic stem cell fate. Oncogene.

[5] Kopp H-G, Avecilla ST, Hooper AT, Rafii S (2005). The bone marrow vascular niche: home of HSC differentiation and mobilization. Physiology (Bethesda).

[6] Wilson A, Trumpp A (2006). Bone-marrow haematopoietic-stem-cell niches. Nat Rev Immunol.

[7] Calvi LM, Adams GB, Weibrecht KW, Weber JM, Olson DP, Knight MC, Martin RP, Schipani E, Divieti P, Bringhurst FR, Milner LA, Kronenberg HM, Scadden DT (2003). Osteoblastic cells regulate the haematopoietic stem cell niche. Nature.

[8] Rizo A, Vellenga E, de Haan G, Schuringa JJ (2006). Signaling pathways in self-renewing hematopoietic and leukemic stem cells: do all stem cells need a niche?. Human molecular genetics.

[9] Zhang J, Niu C, Ye L, Huang H, He X, Tong WG, Ross J, Haug J, Johnson T, Feng JQ, Harris S, Wiedemann LM, Mishina Y, Li L (2003). Identification of the haematopoietic stem cell niche and control of the niche size. Nature.

[10] Abkowitz JL, Robinson AE, Kale S, Long MW, Chen J (2003). Mobilization of hematopoietic stem cells during homeostasis and after cytokine exposure. Blood.

[11] Kopp HG, Avecilla ST, Hooper AT, Rafii S (2005). The bone marrow vascular niche: home of HSC differentiation and mobilization. Physiology.

[12] Yin T, Li L (2006). The stem cell niches in bone. The Journal of clinical investigation.

[13] Ribatti D, Nico B, Vacca A (2006). Importance of the bone marrow microenvironment in inducing the angiogenic response in multiple myeloma. Oncogene.

[14] Ribatti D (2013). Mast cells and macrophages exert beneficial and detrimental effects on tumor progression and angiogenesis. Immunol Lett.

[15] Kalluri R, Zeisberg M (2006). Fibroblasts in cancer. Nat Rev Cancer.

[16] Gabbiani G, Ryan GB, Majne G (1971). Presence of modified fibroblasts in granulation tissue and their possible role in wound contraction. Experientia.

[17] Madar S, Goldstein I, Rotter V (2013). ‘Cancer associated fibroblasts’–more than meets the eye. Trends Mol Med.

[18] Franco OE, Shaw AK, Strand DW, Hayward SW (2010). Cancer associated fibroblasts in cancer pathogenesis. Semin Cell Dev Biol.

[19] Orimo A, Weinberg RA (2006). Stromal fibroblasts in cancer: a novel tumor-promoting cell type. Cell Cycle.

[20] Shimoda M, Mellody KT, Orimo A (2010). Carcinoma-associated fibroblasts are a rate-limiting determinant for tumour progression. Semin Cell Dev Biol.

[21] Erez N, Truitt M, Olson P, Arron ST, Hanahan D (2010). Cancer-Associated Fibroblasts Are Activated in Incipient Neoplasia to Orchestrate Tumor-Promoting Inflammation in an NF-kappaB-Dependent Manner. Cancer Cell.

[22] Orimo A, Gupta PB, Sgroi DC, Arenzana-Seisdedos F, Delaunay T, Naeem R, Carey VJ, Richardson AL, Weinberg RA (2005). Stromal fibroblasts present in invasive human breast carcinomas promote tumor growth and angiogenesis through elevated SDF-1/CXCL12 secretion. Cell.

[23] Cirri P, Chiarugi P (2012). Cancer-associated-fibroblasts and tumour cells: a diabolic liaison driving cancer progression. Cancer Metastasis Rev.

[24] Rasanen K, Vaheri A (2010). Activation of fibroblasts in cancer stroma. Exp Cell Res.

[25] Chen L, Tredget EE, Wu PYG, Wu Y (2008). Paracrine factors of mesenchymal stem cells recruit macrophages and endothelial lineage cells and enhance wound healing. PLoS One.

[26] Fukumura D, Xavier R, Sugiura T, Chen Y, Park EC, Lu N, Selig M, Nielsen G, Taksir T, Jain RK, Seed B (1998). Tumor induction of VEGF promoter activity in stromal cells. Cell.

[27] Byzova TV, Goldman CK, Pampori N, Thomas KA, Bett A, Shattil SJ, Plow EF (2000). A mechanism for modulation of cellular responses to VEGF: activation of the integrins. Mol Cell.

[28] De Wever O, Van Bockstal M, Mareel M, Hendrix A, Bracke M (2014). Carcinoma-associated fibroblasts provide operational flexibility in metastasis. Semin Cancer Biol.

[29] Cirri P, Chiarugi P (2011). Cancer associated fibroblasts: the dark side of the coin. Am J Cancer Res.

[30] Kojima Y, Acar A, Eaton EN, Mellody KT, Scheel C, Ben-Porath I, Onder TT, Wang ZC, Richardson AL, Weinberg RA, Orimo A (2010). Autocrine TGF-beta and stromal cell-derived factor-1 (SDF-1) signaling drives the evolution of tumor-promoting mammary stromal myofibroblasts. Proc Natl Acad Sci U S A.

[31] Radisky DC, Kenny PA, Bissell MJ (2007). Fibrosis and cancer: do myofibroblasts come also from epithelial cells via EMT?. J Cell Biochem.

[32] Weber WA, Petersen V, Schmidt B, Tyndale-Hines L, Link T, Peschel C, Schwaiger M (2003). Positron emission tomography in non-small-cell lung cancer: prediction of response to chemotherapy by quantitative assessment of glucose use. J Clin Oncol.

[33] Kurose K, Gilley K, Matsumoto S, Watson PH, Zhou X-P, Eng C (2002). Frequent somatic mutations in PTEN and TP53 are mutually exclusive in the stroma of breast carcinomas. Nat Genet.

[34] Moinfar F, Man YG, Bratthauer GL, Ratschek M, Tavassoli FA (2000). Genetic abnormalities in mammary ductal intraepithelial neoplasia-flat type (“clinging ductal carcinoma in situ”): a simulator of normal mammary epithelium. Cancer.

[35] Radisky DC, Levy DD, Littlepage LE, Liu H, Nelson CM, Fata JE, Leake D, Godden EL, Albertson DG, Nieto MA, Werb Z, Bissell MJ (2005). Rac1b and reactive oxygen species mediate MMP-3-induced EMT and genomic instability. Nature.

[36] Qiu W, Hu M, Sridhar A, Opeskin K, Fox S, Shipitsin M, Trivett M, Thompson ER, Ramakrishna M, Gorringe KL, Polyak K, Haviv I, Campbell IG (2008). No evidence of clonal somatic genetic alterations in cancer-associated fibroblasts from human breast and ovarian carcinomas. Nat Genet.

[37] Zeisberg EM, Potenta S, Xie L, Zeisberg M, Kalluri R (2007). Discovery of endothelial to mesenchymal transition as a source for carcinoma-associated fibroblasts. Cancer Res.

[38] Kidd S, Spaeth E, Watson K, Burks J, Lu H, Klopp A, Andreeff M, Marini FC (2012). Origins of the tumor microenvironment: quantitative assessment of adipose-derived and bone marrow-derived stroma. PLoS One.

[39] Spaeth EL, Dembinski JL, Sasser AK, Watson K, Klopp A, Hall B, Andreeff M, Marini F (2009). Mesenchymal stem cell transition to tumor-associated fibroblasts contributes to fibrovascular network expansion and tumor progression. PLoS One.

[40] Mishra PJ, Mishra PJ, Humeniuk R, Medina DJ, Alexe G, Mesirov JP, Ganesan S, Glod JW, Banerjee D (2008). Carcinoma-associated fibroblast-like differentiation of human mesenchymal stem cells. Cancer Res.

[41] Barcellos-de-Souza P, Gori V, Bambi F, Chiarugi P (2013). Tumor microenvironment: bone marrow-mesenchymal stem cells as key players. Biochim Biophys Acta.

[42] Uccelli A, Moretta L, Pistoia V (2008). Mesenchymal stem cells in health and disease. Nat Rev Immunol.

[43] Haniffa MA, Collin MP, Buckley CD, Dazzi F (2009). Mesenchymal stem cells: the fibroblasts' new clothes?. Haematologica.

[44] Jones S, Horwood N, Cope A, Dazzi F (2007). The antiproliferative effect of mesenchymal stem cells is a fundamental property shared by all stromal cells. J Immunol.

[45] Tropel P, Noel D, Platet N, Legrand P, Benabid AL, Berger F (2004). Isolation and characterisation of mesenchymal stem cells from adult mouse bone marrow. Experimental cell research.

[46] Dominici M, Le Blanc K, Mueller I, Slaper-Cortenbach I, Marini F, Krause D, Deans R, Keating A, Prockop D, Horwitz E (2006). Minimal criteria for defining multipotent mesenchymal stromal cells. The International Society for Cellular Therapy position statement. Cytotherapy.

[47] Frassanito MA, Rao L, Moschetta M, Ria R, Di Marzo L, De Luisi A, Racanelli V, Catacchio I, Berardi S, Basile A, Menu E, Ruggieri S, Nico B, Ribatti D, Fumarulo R, Dammacco F (2014). Bone marrow fibroblasts parallel multiple myeloma progression in patients and mice: *in vitro* and *in vivo* studies. Leukemia.

[48] Vykoupil KF, Thiele J, Georgii A (1976). Hairy cell leukemia. Bone marrow findings in 24 patients. Virchows Arch A Pathol Anat Histol.

[49] Burthem J, Cawley JC (1994). The bone marrow fibrosis of hairy-cell leukemia is caused by the synthesis and assembly of a fibronectin matrix by the hairy cells. Blood.

[50] Shehata M, Schwarzmeier JD, Hilgarth M, Hubmann R, Duechler M, Gisslinger H (2004). TGF-beta1 induces bone marrow reticulin fibrosis in hairy cell leukemia. J Clin Invest.

[51] Aziz KA, Till KJ, Chen H, Slupsky JR, Campbell F, Cawley JC, Zuzel M (2003). The role of autocrine FGF-2 in the distinctive bone marrow fibrosis of hairy-cell leukemia (HCL). Blood.

[52] Blau O, Baldus CD, Hofmann W-K, Thiel G, Nolte F, Burmeister T, Turkmen S, Benlasfer O, Schumann E, Sindram A, Molkentin M, Mundlos S, Keilholz U, Thiel E, Blau IW (2011). Mesenchymal stromal cells of myelodysplastic syndrome and acute myeloid leukemia patients have distinct genetic abnormalities compared with leukemic blasts. Blood.

[53] Menendez P, Catalina P, Rodriguez R, Melen GJ, Bueno C, Arriero M, Garcia-Sanchez F, Lassaletta A, Garcia-Sanz R, Garcia-Castro J (2009). Bone marrow mesenchymal stem cells from infants with MLL-AF4+ acute leukemia harbor and express the MLL-AF4 fusion gene. J Exp Med.

[54] Shalapour S, Eckert C, Seeger K, Pfau M, Prada J, Henze G, Blankenstein T, Kammertoens T (2010). Leukemia-associated genetic aberrations in mesenchymal stem cells of children with acute lymphoblastic leukemia. J Mol Med.

[55] Hida K, Hida Y, Amin DN, Flint AF, Panigrahy D, Morton CC, Klagsbrun M (2004). Tumor-associated endothelial cells with cytogenetic abnormalities. Cancer Res.

[56] Gluck U, Zipori D, Wetzler M, Berrebi A, Shaklai M, Drezen O, Zaizov R, Luria D, Marcelle C, Stark B (1989). Long-term proliferation of human leukemia cells induced by mouse stroma. Exp Hemaol.

[57] Kumagai M, Manabe A, Pui CH, Behm FG, Raimondi SC, Hancock ML, Mahmoud H, Crist WM, Campana D (1996). Stroma-supported culture in childhood B-lineage acute lymphoblastic leukemia cells predicts treatment outcome. J Clin Invest.

[58] Makrynikola V, Bradstock KF (1993). Adhesion of precursor-B acute lymphoblastic leukaemia cells to bone marrow stromal proteins. Leukemia.

[59] Manabe A, Coustan-Smith E, Behm FG, Raimondi SC, Campana D (1992). Bone marrow-derived stromal cells prevent apoptotic cell death in B-lineage acute lymphoblastic leukemia. Blood.

[60] Nishigaki H, Ito C, Manabe A, Kumagai M, Coustan-Smith E, Yanishevski Y, Behm FG, Raimondi SC, Pui CH, Campana D (1997). Prevalence and growth characteristics of malignant stem cells in B-lineage acute lymphoblastic leukemia. Blood.

[61] Umiel T, Friedman S, Zaizov R, Cohen IJ, Gozes Y, Epstein N, Kobiler D, Zipori D (1986). Long-term culture of infant leukemia cells: dependence upon stromal cells from the bone marrow and bilineage differentiation. Leuk Res.

[62] Ramasamy R, Lam EWF, Soeiro I, Tisato V, Bonnet D, Dazzi F (2007). Mesenchymal stem cells inhibit proliferation and apoptosis of tumor cells: impact on *in vivo* tumor growth. Leukemia.

[63] Nwabo Kamdje AH, Mosna F, Bifari F, Lisi V, Bassi G, Malpeli G, Ricciardi M, Perbellini O, Scupoli MT, Pizzolo G, Krampera M (2011). Notch-3 and Notch-4 signaling rescue from apoptosis human B-ALL cells in contact with human bone marrow-derived mesenchymal stromal cells. Blood.

[64] Iwamoto S, Mihara K, Downing JR, Pui C-H, Campana D (2007). Mesenchymal cells regulate the response of acute lymphoblastic leukemia cells to asparaginase. J Clin Invest.

[65] Ehsanipour EA, Sheng X, Behan JW, Wang X, Butturini A, Avramis VI, Mittelman SD (2013). Adipocytes cause leukemia cell resistance to L-asparaginase via release of glutamine. Cancer Res.

[66] Vianello F, Villanova F, Tisato V, Lymperi S, Ho K-K, Gomes AR, Marin D, Bonnet D, Apperley J, Lam EWF, Dazzi F (2010). Bone marrow mesenchymal stromal cells non-selectively protect chronic myeloid leukemia cells from imatinib-induced apoptosis via the CXCR4/CXCL12 axis. Haematologica.

[67] Jin L, Tabe Y, Konoplev S, Xu Y, Leysath CE, Lu H, Kimura S, Ohsaka A, Rios M-B, Calvert L, Kantarjian H, Andreeff M, Konopleva M (2008). CXCR4 up-regulation by imatinib induces chronic myelogenous leukemia (CML) cell migration to bone marrow stroma and promotes survival of quiescent CML cells. Mol Cancer Ther.

[68] Pillozzi S, Masselli M, De Lorenzo E, Accordi B, Cilia E, Crociani O, Amedei A, Veltroni M, D'Amico M, Basso G, Becchetti A, Campana D, Arcangeli A (2011). Chemotherapy resistance in acute lymphoblastic leukemia requires hERG1 channels and is overcome by hERG1 blockers. Blood.

[69] Yang Y, Mallampati S, Sun B, Zhang J, Kim S-B, Lee J-S, Gong Y, Cai Z, Sun X (2013). Wnt pathway contributes to the protection by bone marrow stromal cells of acute lymphoblastic leukemia cells and is a potential therapeutic target. Cancer Lett.

[70] Greaves MF, Wiemels J (2003). Origins of chromosome translocations in childhood leukaemia. Nat Rev Cancer.

[71] Conforti A, Biagini S, Del Bufalo F, Sirleto P, Angioni A, Starc N, Li Pira G, Moretta F, Proia A, Contoli B, Genovese S, Ciardi C, Avanzini MA, Rosti V, Lo-Coco F, Locatelli F (2013). Biological, functional and genetic characterization of bone marrow-derived mesenchymal stromal cells from pediatric patients affected by acute lymphoblastic leukemia. PLoS One.

[72] Campioni D, Bardi MA, Cavazzini F, Tammiso E, Pezzolo E, Pregnolato E, Volta E, Cuneo A, Lanza F (2012). Cytogenetic and molecular cytogenetic profile of bone marrow-derived mesenchymal stromal cells in chronic and acute lymphoproliferative disorders. Ann Hematol.

[73] Raaijmakers MHGP, Mukherjee S, Guo S, Zhang S, Kobayashi T, Schoonmaker JA, Ebert BL, Al-Shahrour F, Hasserjian RP, Scadden EO, Aung Z, Matza M, Merkenschlager M, Lin C, Rommens JM, Scadden DT (2010). Bone progenitor dysfunction induces myelodysplasia and secondary leukaemia. Nature.

[74] Tashiro H, Mizutani-Noguchi M, Shirasaki R, Shirafuji N (2010). Acute myelogenous leukemia cells with the MLL-ELL translocation convert morphologically and functionally into adherent myofibroblasts. Biochem Biophys Res Commun.

[75] Avanzini MA, Bernardo ME, Novara F, Mantelli M, Poletto V, Villani L, Lenta E, Ingo DM, Achille V, Bonetti E, Massa M, Campanelli R, Fois G, Catarsi P, Gale RP, Moretta A (2014). Functional and genetic aberrations of *in vitro*-cultured marrow-derived mesenchymal stromal cells of patients with classical Philadelphia-negative myeloproliferative neoplasms. Leukemia.

[76] Lagneaux L, Delforge A, Bron D, De Bruyn C, Stryckmans P (1998). Chronic lymphocytic leukemic B cells but not normal B cells are rescued from apoptosis by contact with normal bone marrow stromal cells. Blood.

[77] Panayiotidis P, Jones D, Ganeshaguru K, Foroni L, Hoffbr AV (1996). Human bone marrow stromal cells prevent apoptosis and support the survival of chronic lymphocytic leukaemia cells *in vitro*. Br J Haematol.

[78] Ding W, Knox TR, Tschumper RC, Wu W, Schwager SM, Boysen JC, Jelinek DF, Kay NE (2010). Platelet-derived growth factor (PDGF)-PDGF receptor interaction activates bone marrow-derived mesenchymal stromal cells derived from chronic lymphocytic leukemia: implications for an angiogenic switch. Blood.

[79] Zhang W, Trachootham D, Liu J, Chen G, Pelicano H, Garcia-Prieto C, Lu W, Burger JA, Croce CM, Plunkett W, Keating MJ, Huang P (2012). Stromal control of cystine metabolism promotes cancer cell survival in chronic lymphocytic leukaemia. Nat Cell Biol.

[80] Lutzny G, Kocher T, Schmidt-Supprian M, Rudelius M, Klein-Hitpass L, Finch AJ, Durig J, Wagner M, Haferlach C, Kohlmann A, Schnittger S, Seifert M, Wanninger S, Zaborsky N, Oostendorp R, Ruland J (2013). Protein kinase c-beta-dependent activation of NF-kappaB in stromal cells is indispensable for the survival of chronic lymphocytic leukemia B cells *in vivo*. Cancer Cell.

[81] Balakrishnan K, Burger JA, Quiroga MP, Henneberg M, Ayres ML, Wierda WG, Ghi V (2010). Influence of bone marrow stromal microenvironment on forodesine-induced responses in CLL primary cells. Blood.

[82] Jundt F, Anagnostopoulos I, Bommert K, Emmerich F, Muller G, Foss HD, Royer HD, Stein H, Dorken B (1999). Hodgkin/Reed-Sternberg cells induce fibroblasts to secrete eotaxin, a potent chemoattractant for T cells and eosinophils. Blood.

[83] Aldinucci D, Poletto D, Nanni P, Degan M, Gloghini A, Di Francia R, Russo S, Carbone A, Pinto A, Gattei V (2002). Hodgkin and Reed-Sternberg cells express functional c-kit receptors and interact with primary fibroblasts from Hodgkin's disease-involved lymph nodes through soluble and membrane-bound stem cell factor. Br J Haematol.

[84] Cattaruzza L, Gloghini A, Olivo K, Di Francia R, Lorenzon D, De Filippi R, Carbone A, Colombatti A, Pinto A, Aldinucci D (2009). Functional coexpression of Interleukin (IL)-7 and its receptor (IL-7R) on Hodgkin and Reed-Sternberg cells: Involvement of IL-7 in tumor cell growth and microenvironmental interactions of Hodgkin's lymphoma. Int J Cancer.

[85] Mueller SN, Germain RN (2009). Stromal cell contributions to the homeostasis and functionality of the immune system. Nat Rev Immunol.

[86] Thomazy VA, Vega F, Medeiros LJ, Davies PJ, Jones D (2003). Phenotypic modulation of the stromal reticular network in normal and neoplastic lymph nodes: tissue transglutaminase reveals coordinate regulation of multiple cell types. Am J Pathol.

[87] Chang K-C, Huang X, Medeiros LJ, Jones D (2003). Germinal centre-like versus undifferentiated stromal immunophenotypes in follicular lymphoma. J Pathol.

[88] Escudier B, Porta C, Bono P, Powles T, Eisen T, Sternberg CN, Gschwend JE, De Giorgi U, Parikh O, Hawkins R, Sevin E, Negrier S, Khan S, Diaz J, Redhu S, Mehmud F (2014). Randomized, controlled, double-blind, cross-over trial assessing treatment preference for pazopanib versus sunitinib in patients with metastatic renal cell carcinoma: PISCES Study. J Clin Oncol.

[89] Rosen PJ, Sweeney CJ, Park DJ, Beaupre DM, Deng H, Leitch IM, Shubhakar P, Zhu M, Oliner KS, Anderson A, Yee LK (2010). A phase Ib study of AMG 102 in combination with bevacizumab or motesanib in patients with advanced solid tumors. Clin Cancer Res.

[90] Ebos JML, Kerbel RS (2011). Antiangiogenic therapy: impact on invasion, disease progression, and metastasis. Nat Rev Clin Oncol.

[91] Kim KJ, Wang L, Su Y-C, Gillespie GY, Salhotra A, Lal B, Laterra J (2006). Systemic anti-hepatocyte growth factor monoclonal antibody therapy induces the regression of intracranial glioma xenografts. Clin Cancer Res.

[92] Pietras K, Pahler J, Bergers G, Hanahan D (2008). Functions of paracrine PDGF signaling in the proangiogenic tumor stroma revealed by pharmacological targeting. PLoS Med.

[93] Wen J, Matsumoto K, Taniura N, Tomioka D, Nakamura T (2004). Hepatic gene expression of NK4, an HGF-antagonist/angiogenesis inhibitor, suppresses liver metastasis and invasive growth of colon cancer in mice. Cancer Gene Ther.

[94] Smith HW, Marshall CJ (2010). Regulation of cell signalling by uPAR. Nat Rev Mol Cell Biol.

[95] Bene MC, Castoldi G, Knapp W, Rigolin GM, Escribano L, Lemez P, Ludwig WD, Matutes E, Orfao A, Lanza F, van't Veer M (2004). CD87 (urokinase-type plasminogen activator receptor), function and pathology in hematological disorders: a review. Leukemia.

[96] Jacobsen B, Ploug M (2008). The urokinase receptor and its structural homologue C4.4A in human cancer: expression, prognosis and pharmacological inhibition. Curr Med Chem.

[97] Ulisse S, Baldini E, Sorrenti S, D'Armiento M (2009). The urokinase plasminogen activator system: a target for anti-cancer therapy. Curr Cancer Drug Targets.

[98] Greenhough A, Smartt HJM, Moore AE, Roberts HR, Williams AC, Paraskeva C, Kaidi A (2009). The COX-2/PGE2 pathway: key roles in the hallmarks of cancer and adaptation to the tumour microenvironment. Carcinogenesis.

[99] Sato N, Maehara N, Goggins M (2004). Gene expression profiling of tumor-stromal interactions between pancreatic cancer cells and stromal fibroblasts. Cancer Res.

[100] Giannoni E, Bianchini F, Masieri L, Serni S, Torre E, Calorini L, Chiarugi P (2010). Reciprocal activation of prostate cancer cells and cancer-associated fibroblasts stimulates epithelial-mesenchymal transition and cancer stemness. Cancer Res.

[101] Ghosh N, Chaki R, Mandal V, Mandal SC (2010). COX-2 as a target for cancer chemotherapy. Pharmacol Rep.

[102] Chen L, Tredget EE, Wu PY, Wu Y (2008). Paracrine factors of mesenchymal stem cells recruit macrophages and endothelial lineage cells and enhance wound healing. PLoS One.

[103] Chaudhary AK, Singh M, Bharti AC, Asotra K, Sundaram S, Mehrotra R (2010). Genetic polymorphisms of matrix metalloproteinases and their inhibitors in potentially malignant and malignant lesions of the head and neck. J Biomed Sci.

[104] Liu R, Li H, Liu L, Yu J, Ren X (2012). Fibroblast activation protein: A potential therapeutic target in cancer. Cancer Biol Ther.

[105] Scott AM, Wiseman G, Welt S, Adjei A, Lee F-T, Hopkins W, Divgi CR, Hanson LH, Mitchell P, Gansen DN, Larson SM, Ingle JN, Hoffman EW, Tanswell P, Ritter G, Cohen LS (2003). A Phase I dose-escalation study of sibrotuzumab in patients with advanced or metastatic fibroblast activation protein-positive cancer. Clin Cancer Res.

[106] Hofheinz RD, Weisser A, Willer A, Hehlmann R, Hochhaus A (2003). Treatment of a patient with advanced esophageal cancer with a combination of mitomycin C and capecitabine: activation of the thymidine phosphorylase as active principle?. Onkologie.

[107] Ostermann E, Garin-Chesa P, Heider KH, Kalat M, Lamche H, Puri C, Kerjaschki D, Rettig WJ, Adolf GR (2008). Effective immunoconjugate therapy in cancer models targeting a serine protease of tumor fibroblasts. Clin Cancer Res.

[108] Desgrosellier JS, Cheresh DA (2010). Integrins in cancer: biological implications and therapeutic opportunities. Nat Rev Cancer.

[109] Zhuang Z, Zhou R, Xu X, Tian T, Liu Y, Liu Y, Lian P, Wang J, Xu K (2013). Clinical significance of integrin αvβ6 expression effects on gastric carcinoma invasiveness and progression via cancer-associated fibroblasts. Med Oncol.

[110] Eberlein C, Kendrew J, McDaid K, Alfred A, Kang JS, Jacobs VN, Ross SJ, Rooney C, Smith NR, Rinkenberger J, Cao A, Churchman A, Marshall JF, Weir HM, Bedian V, Blakey DC (2013). A human monoclonal antibody 264RAD targeting αvβ6 integrin reduces tumour growth and metastasis, and modulates key biomarkers *in vivo*. Oncogene.

[111] Dudas J, Fullar A, Romani A, Pritz C, Kovalszky I, Hans Schartinger V, Mathias Sprinzl G, Riechelmann H (2013). Curcumin targets fibroblast-tumor cell interactions in oral squamous cell carcinoma. Exp Cell Res.

